# Differential Effects of Natural Grazing and Feedlot Feeding on Yak Fecal Microbiota

**DOI:** 10.3389/fvets.2022.791245

**Published:** 2022-04-15

**Authors:** Tariq Shah, Luming Ding, Ahmad Ud Din, Faiz-ul Hassan, Anum Ali Ahmad, Haiyan Wei, Xianju Wang, Qi Yan, Muhammad Ishaq, Niyaz Ali, Yougui Fang

**Affiliations:** ^1^State Key Laboratory of Grassland Agro-Ecosystem, College of Ecology, Lanzhou University, Lanzhou, China; ^2^Qinghai Provincial Key Laboratory of Adaptive Management on Alpine Grassland, Qinghai University, Xining, China; ^3^Drug Discovery Research Center, Southwest Medical University, Luzhou, China; ^4^Institutes for Systems Genetics, Frontiers Science Center for Disease-related Molecular Network, West China Hospital, Sichuan University, Chengdu, China; ^5^Faculty of Animal Husbandry, Institute of Animal and Dairy Sciences, University of Agriculture, Faisalabad, Pakistan; ^6^School of Life Sciences, Probiotics and Biological Feed Research Centre, Lanzhou University, Lanzhou, China; ^7^State Key Laboratory of Subtropical Agro Bio–Resource and College of Life Sciences, Guangxi University, Nanning, China; ^8^Key Laboratory of Adaptation and Evolution of Plateau Biota, Northwest Institute of Plateau Biology, Chinese Academy of Sciences, Xining, China

**Keywords:** feedlot, fecal microbiota, grazing (rangelands), bacterial diversity, yak

## Abstract

Variation in food and diet shapes the diversity of the gut microbiota of ruminants. The present study investigated the microbial diversity in the fecal microbiota of yaks reared under natural grazing and feedlot system. A total of 48 domestic yaks with an average age of 7.5 years were selected from two different grazing habitats: one group grazed on natural pasture (grazing yaks—GY) while the other group was fed fodder and concentrate (feedlot yaks—FY). Crude protein, non-fiber carbohydrate, hemicelluloses, and digestible dry matter contents of natural pastures were higher than those in the feedlot. The lower insoluble fiber contents were found in grazing land. The 16S rRNA gene sequencing revealed 675 and 348 unique operational taxonomic units (OTUs) in the GY and FY, respectively, in addition to 1,778 common OTUs. Overall, a total of 9,891 OTUs were identified as a whole, of which 6,160 OTUs were from GY and 3,731 were from FY. Shannon index analysis revealed a higher bacterial diversity in GY than FY. At the phylum level, Firmicutes were dominant bacterial taxa in both groups. The relative abundance of Firmicutes in GY (56% ± 0.05) was higher than in FY (41% ± 0.08). At the family level, GY had a significantly higher abundance of Ruminococcaceae (*p* < 0.001) and Rikenellaceae (*p* < 0.001) than FY, but FY had a significantly higher abundance of Prevotellaceae than GY (*p* < 0.001). At the genus level, abundances of *Faecalibacterium, Alloprevotella*, and *Succinivibrio* were higher in FY than in GY. This study presents novel information on fecal bacterial composition and diversity in yaks reared under two different production systems.

## Introduction

The yak (*Poephagus grunniens*) is well-adapted to the harsh environment of the high-altitude Qinghai Tibetan Plateau (QTP), which is characterized by low air oxygen content, strong ultraviolet radiation, low air temperatures, and sparse forage resources of often poor quality (1). Dietary protein and energy requirements for maintenance are comparatively lower in Yaks with a higher efficiency of microbial protein synthesis (2). Yaks provide meat, milk, transportation, and draft power for the local herders. Moreover, yak milk is also important for health maintenance, because it contains polyunsaturated fatty acid such as conjugated linoleic acid and essential minerals like calcium and phosphorus (3). Caseins in yak milk have proved antihypertensive activity (4). In Tibet, Nepal, some parts of Mongolia, and China, yak milk is widely used for human consumption (3). Therefore, yaks' production has gained attention owing to its multipurpose utility and as a source of livelihood for local farmers.

Similar to other ruminants, yak rumen also inhabits anaerobic bacteria, fungi, methanogens, and ciliated protozoa, which play an important role in feed digestion and microbial protein synthesis (2, 5). Therefore, balanced rumen microbiota plays a crucial role in maintaining the stability of the rumen environment and the health of the host animal (6, 7). Moreover, microbial composition and stability are also essential for the ruminant's welfare, health, and production efficiency (7). The predominant bacterial phyla recorded in yak rumen are Bacteroidetes and Firmicutes, accounting for ~80% of the total reads, with low abundances (<10%) of Fibrobacter, Spirochaeta, and Proteobacteria (8). However, the dominance of Bacteroidetes and Firmicutes for different yaks can differ (8, 9). Under grazing conditions, in one study, 23 phyla having 159 families were identified in yak rumen fluid in which Firmicutes dominated with 46%, followed by Bacteroidetes with 40% (9), whereas in another study, Bacteroidetes dominated with 52%, followed by Firmicutes with 34% (8). Although Bacteroidetes and Firmicutes were predominant bacterial phyla under the grazing conditions in both studies, but species diversity and richness were substantially different.

Rumen microbial compositions and variations are important not only for the health and production of ruminants but also for reducing methane (CH_4_) emission (10). Factors including diet, age, species, and seasonal variations affect the ruminal microbiota, with diet being the most important factor (9). Rumen microbe's efficiency and the rumen microbial community composition is associated with diet and feeding patterns (11). Diverse rumen microbial diversity was observed in yaks with different indoor or grazing feeding patterns (12). Under grazing conditions, the microbial diversity of yaks feeding on the re-green stage was higher than yaks feeding on the grassy and withered stages (8). The rumen prokaryotic diversity of yaks while grazing natural pasture was higher than their lowland counterparts fed a total mixed ratio (13). Therefore, microbe diversity is linked to nutrient quality, feeding system, geographical location, and environment. With the increasing burden of environmental and efficiency requirements, more and more yak fattening is done in confinement. Moreover, yak raising is also becoming popular in lower-elevation agricultural areas with good feed resources. The abrupt change of feed (from pasture forage to concentrate) and environment (from pasture to feedlot) will cause the shift in rumen environment of yak. Therefore, the current study aimed to investigate and compare the fecal microbiota of yaks traditionally grazing in the pasture and confinement with high concentrate feeding. The objective was to reveal the change of yak fecal microbiota from grazing to confinement and evaluate the new fattening management practice for yaks.

## Materials and Methods

### The Experimental Site, Animals, and Management

For the grazing yaks (GY), fresh fecal samples were collected from grazing site at Qinghai Datong yak breeding farm (elevation, 3,200 m), while for feedlot yak (FY), fresh fecal samples were collected at Gansu Dehua yak fattening farm (elevation, 2,300 m) during October 2017. A total of 48 fecal samples were collected (24 from each farm site). The fresh fecal samples were collected immediately after defecation. The samples were taken from the uppermost parts of the piles carefully to avoid soil contamination. The fecal samples were temporarily stored in an icebox and transferred immediately to a −80°C freezer. The group of grazing yaks (GY) was selected from a herd of 200 animals that only grazed in the natural alpine pasture from 7 a.m. to 6 p.m. in the daytime. The pasture was dominated by herbage species of *Kobresia humilis, Kobresia graminifolia, Elymus nutan, Kobresia pygmaea, Anaphalis lacteal, Polyginum viviparum, Potentilla fruticose, Cortaderia jubata*, and *Sibiraea angustata*. These yaks were confined in a compartment at night. The fattening yaks (FY) from a herd of 1,000 yaks were selected that were fed a high concentrate diet with 90% concentrate and 10% roughage. The FY yaks were provided feed in compartments two times, i.e., 7 a.m. and 4 p.m., respectively. The drinking water was available *ad libitum* for both groups.

### Determination of Nutritional Composition of Experimental Forages

Nutrient composition of forages was analyzed for dry matter (DM), organic matter (OM), ash content, crude protein (CP), and ether extract (EE) by the method described by the Association of Analytical Chemists (AOAC) (1990) (14). Neutral detergent fiber (NDF) and acid detergent fiber (ADF) were analyzed according to the method of Goering and Van Soest (1970) (15). The total carbohydrate (TC), non-fiber carbohydrate (NFC), and digestible dry matter (DDM) in experimental forages were calculated as TC = 100 – (CP + EE + Ash), NFC = 100 – (NDF + CP + EE + Ash), DDM (%) = 88.9 – 0.779 ADF and Hemicellulose = NDF –ADF.

### DNA Extraction and MiSeq Sequencing of 16S rRNA Gene Amplicons

Fecal samples were homogenized thoroughly before DNA extraction. Genomic DNA was extracted from a 1-ml mixture of 0.211–0.299 mg of feces and VXL buffer using a bead beater (Mini-bead Beater, Bio Spec Products, Bartlesville, UK). DNA was then extracted by using the QIAamp Fast DNA Stool Mini Kit (Qiagen, Hilden, Germany). The DNA quantity and quality were checked using NanoDrop 2000 (Thermo Scientific, Wilmington, USA), and DNA samples were diluted to 80 ng/μl before being subjected to PCR amplification.

The PCR amplification of bacterial V3–V4 hypervariable region of 16S rRNA gene was performed with universal primers 338F (5′-ACTCCTACGGGAGGCAGCAG-3′) and 806R (5′-GGACTACHVGGGTWTCTAAT-3′) (16). The PCR amplification conditions used were as follows: initial denaturation at 94°C for 3 min, followed by 30 cycles of 94°C for 40 s, 56°C for 60 s, and 72°C for 60 s, and a final extension at 72°C for 10 min. The PCR products were gel purified using GeneJET Gel Recovery Kit (Thermo Scientific, USA) according to the manufacturer's instructions. The purified amplicons were used for the library construction, and sequencing was performed by using an Illumina MiSeq system with the MiSeq Reagent Kit v2 2 × 250 bp (Illumina, San Diego, USA).

### Sequence Data Analysis

The raw sequences were analyzed using QIIME pipeline–version 1.7.0 (http://qiime.org/tutorials/tutorial.html). Chimera sequences were removed using UCHIME algorithm in USEARCH (17). After removing singletons and performing quality control, optimized sequence reads were aligned against the SILVA database, Release128 (http://www.arb-silva.de) for identification of Operational Taxonomic Units (OTU) using cluster identity threshold of 97% sequence similarity as reported previously (18, 19). Bacterial diversity was determined in different treatment groups by analyzing alpha and beta diversity indices from the complete OTU table. Bacterial richness and evenness in each sample were analyzed by measuring Chao and abundance-based coverage estimator (ACE) while alpha diversity was estimated by determining Shannon and Simpson indices (20–23) using QIIME software (Version 1.9.1, http://qiime.org/). Microbial evenness within each sample was assessed by Simpson and Shannon's evenness (Pielou's J) indices (24). At the same time, PCoA and UPGMA were constructed based on the weighted uniFrac distance in RDP (http://pyro.cme.msu.edu/) to evaluate the overall structural changes of fecal bacterial communities. Using the program “Venn Diagram” in R, the OTUs shared between grazing and confined yaks were calculated.

### Statistical Analysis

The Student's *t*-test was used to compare the nutrient composition of natural pasture and forage grasses in SPSS 16.0 software (SPSS Inc., Chicago, IL, USA). Relative abundances of bacterial phyla, families, and genera as well as alpha diversity were compared using the Wilcoxon's ranked test with a false discovery rate (FDR) correction. The linear discriminant analysis effect size (LEfSe) method was used to examine differences at taxonomic levels using an LDA score equal to 4 as a threshold value. A significant difference was considered at *p* < 0.05.

## Results

The chemical compositions of forages in grazing pasture and feedlot are presented in Table 1. The dry matter contents of forages available to both groups (GY and FY) were the same. However, ash, crude protein, ether extract, non-fiber carbohydrate, hemicellulose, and digestible dry matter contents of forages in the pasture were significantly (*p* < 0.05) higher than forages in the feedlot. The OM, insoluble dietary fiber such as NDF and ADF contents, and TC content of forages in the feedlot were significantly (*p* < 0.05) higher than in forages in the pasture.

**Table 1:** The chemical compositions of forages in natural grazing pasture and feedlot.

**Parameters**	**Forage in GY**	**Forage in FY**	***P*-value**
Dry matter	97.0	96.3	0.175
Organic matter	92.70^b^	93.60^a^	0.016
Ash	7.29^a^	6.43b	0.016
Crude protein	15.31^a^	7.25b	0.001
Neutral detergent fiber	53.70^b^	66.70^a^	0.001
Acid detergent fiber	27.70^b^	53.80^a^	0.001
Ether extract	1.36^a^	0.99^b^	0.045
Total carbohydrates	76.40^b^	85.20^a^	0.001
Non-fiber carbohydrates	22.70^a^	18.50^b^	0.013
Hemicellulose	26.0^a^	12.90^b^	0.001

*Values with different superscripts within the same row are significantly different from each other (p < 0.05)*.

### Analysis of Fecal Microbial Diversity

A total of 9,891 OTUs were identified, of which 6,160 were observed in GY and 3,731 were observed in FY. As shown in the Venn diagram, there were 675 unique OTUs in the GY group, and 348 in the FY group, while 1,778 OTUs were common between both groups (Supplementary Figure 1). Moreover, observed species were saturated, and the rarefaction curve of every sample entered the plateau phase. From the rarefaction curve at 97% sequence similarity level of the index of different models, it was observed that the sample number, abundance, and evenness of the intestinal microbial species had met the sequencing and analysis requirements, showing that maximum depth of sequencing had been achieved (Supplementary Figure 2).

### Alpha Diversity

**Figure 1:**
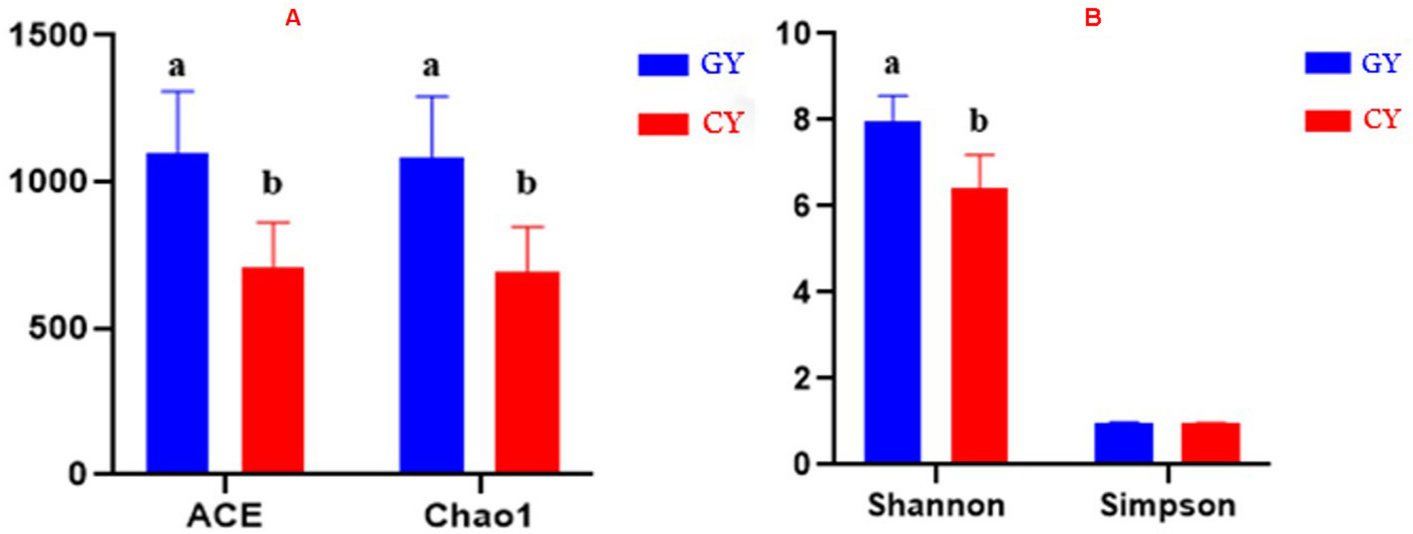
**(A)** ACE and Chao1 indices of bacterial diversity. **(B)** Shannon and Simpson indices of bacterial diversity. GY, Yaks grazing natural pasture; FY, Yaks in feedlots. Bars with different superscripts indicate that mean values are significantly different from each other (*p* < 0.001).

The alpha diversity was assessed using the parameters like Abundance-based Coverage Estimator (ACE), Chao1 index, Shannon index, and Simpson index. ACE and Chao1 index values 1,097.23 ± 211.53 and 1,082.75 ± 209.64, respectively, were higher at *p* < 0.001 for the GY than those of FY ACE and Chao1 values 708.96 ± 153.50 and 692.88 ± 153.05, respectively. The Shannon index (7.98 ± 0.58) was higher (*p* < 0.001) in GY than for FY (6.41 ± 0.78), whereas the Simpson index did not differ (*p* > 0.05) between GY (0.98 ± 0.01) and FY (0.95 ± 0.03) as presented in Figures 1A,B.

### Beta Diversity

**Figure 2:**
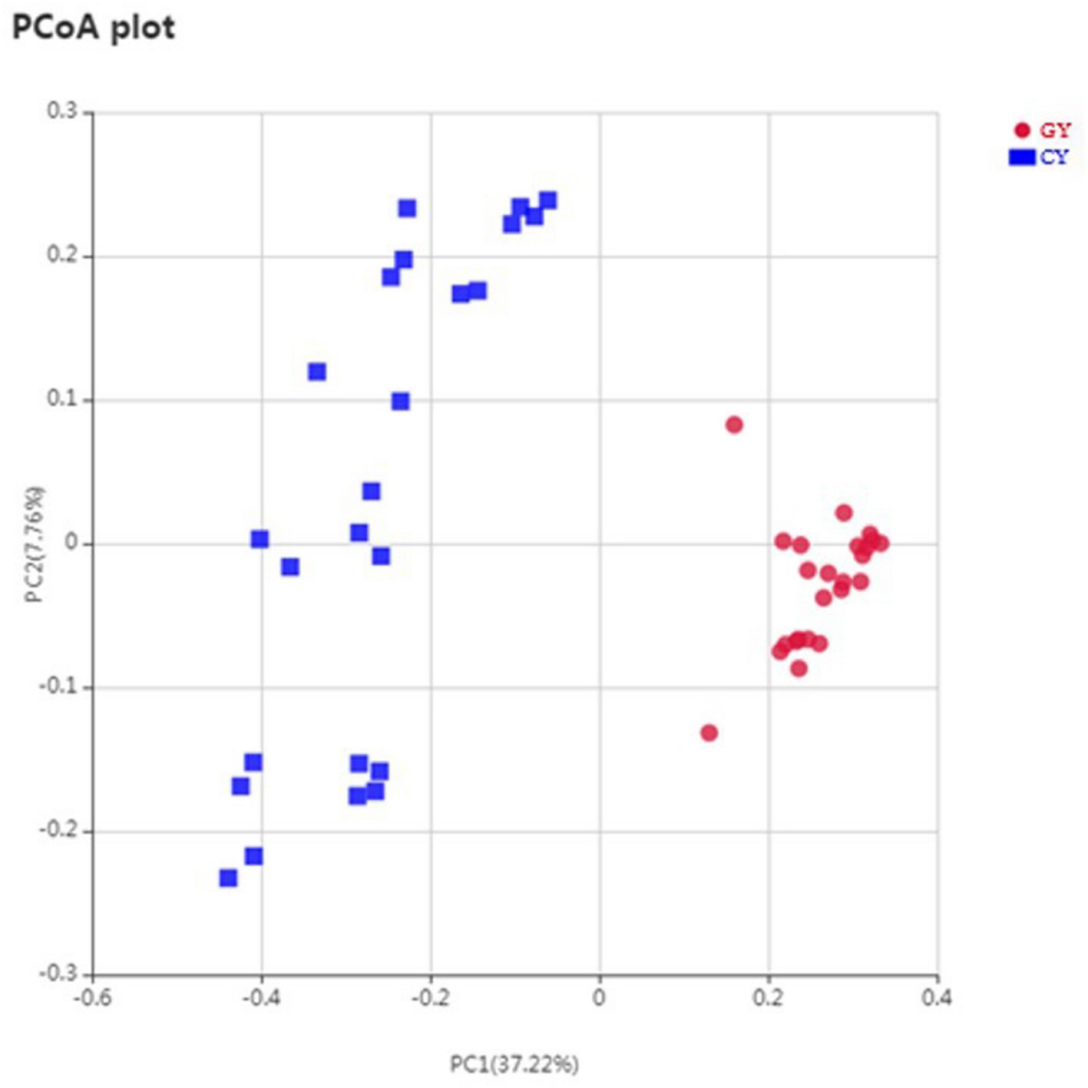
Principal coordinates plot of OTU-level weighted UniFrac distance among grazing (GY) and feedlots (FY) yak groups.

**Figure 3:**
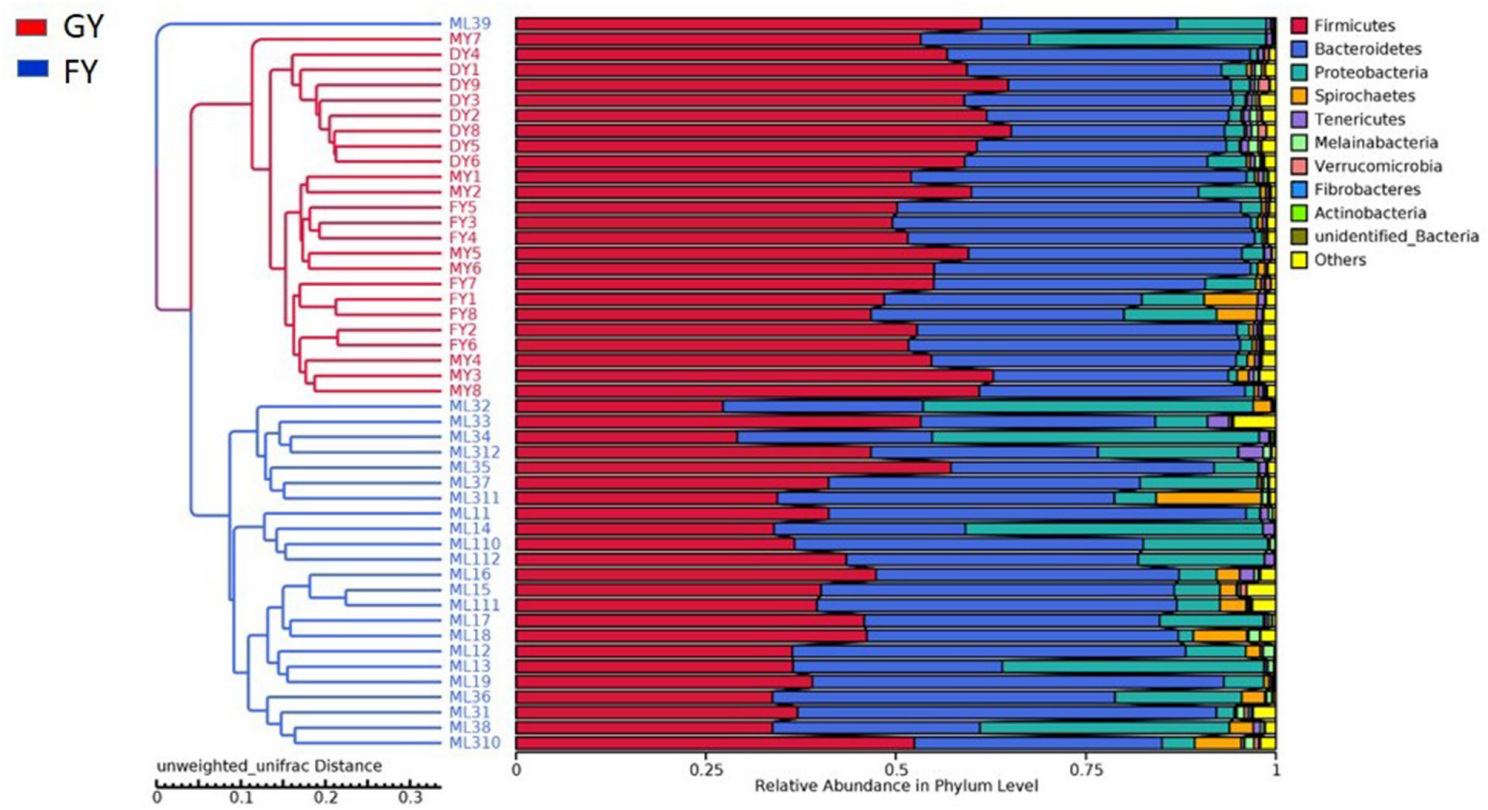
Unweighted pair group method with arithmetic mean (UPGMA) based on the weighted uniFrac distance was used for the clustering analysis of GY and FY group samples. GY, Pasture grazing yaks; FY, feedlot yak group.

Weighted UniFrac metric was used to calculate the dissimilarity in the bacterial community between the GY and FY. The average dissimilarity value in the bacterial community between GY and FY was about 20.4% in GY and 45.2% in FY. According to the Anosim analysis, bacterial community differences and dissimilarity of microbiota in FY were significantly higher (*p* < 0.001) than that of microbiota in GY (Figure 2). The Unweighted pair group method with arithmetic mean (UPGMA) was used for the clustering analysis of the samples belonging to GY and FY (Figure 3). The total classified phyla were about 97.4 and 96.3% of the total fecal microbiota in GY in FY, respectively, with the dominant bacteria phyla including Firmicutes, Bacteroidetes, Proteobacteria, and Spirochaetes. The bacterial phyla, including Tenericutes, Melainabacteria, Verrucomicrobia, Fibrobacteres, Actinobacteria, unknown bacteria, and other phyla with lower count, showed no difference (*p* > 0.05) in relative abundance between GY and FY.

### Relative Abundance of Bacterial Phyla

**Figure 4:**
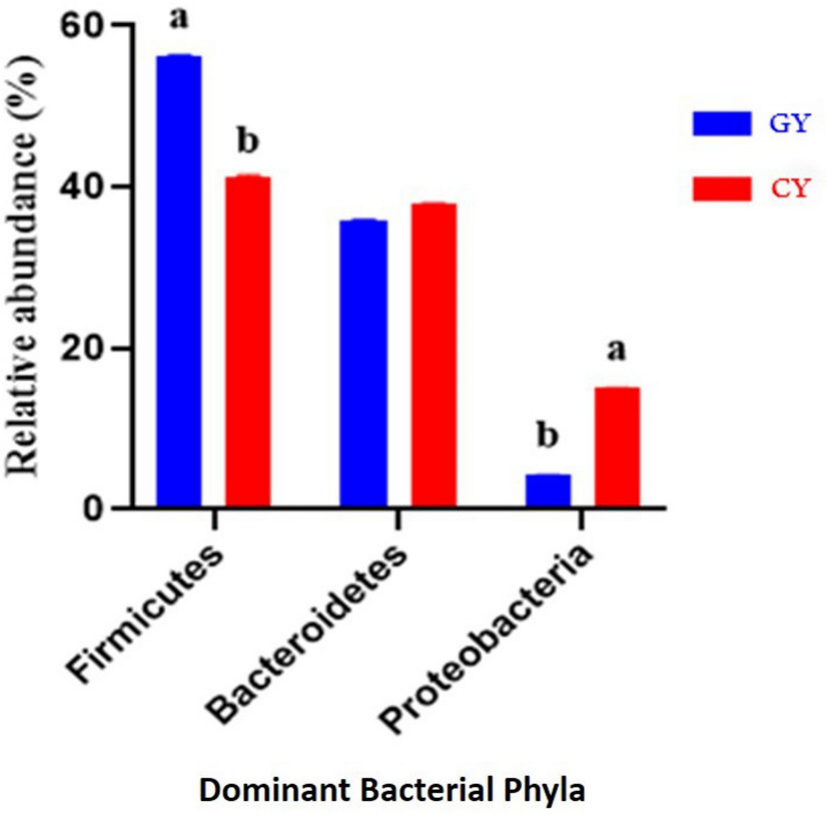
Major bacterial phyla found in yaks fed on natural grazing pasture (GY) and feedlots (FY). Bars with different superscripts indicate that mean values are significantly different from each other (*p* < 0.001).

At the phylum level, there were 21 bacterial phyla identified in GY and FY. The dominant bacterial phyla in GY were Firmicutes (56.3% ± 0.05), Bacteroidetes (35.9% ± 0.07), and Proteobacteria (4.26% ± 0.06), whereas in FY, the dominant phyla were Firmicutes (41.4% ± 0.08), Bacteroidetes (37.9% ± 0.10), and Proteobacteria (15.0% ± 0.13) (Figure 4). Firmicutes in GY were significantly higher (*p* < 0.001) than in FY, but Proteobacteria in FY were significantly higher (*p* < 0.001) than in GY. Melainabacteria, Lentisphaerae, and Verrucomicrobia were less abundant phyla, and their relative abundance in GY was significantly higher (*p* < 0.001) than in FY.

### Relative Abundance of Bacterial Families

**Figure 5:**
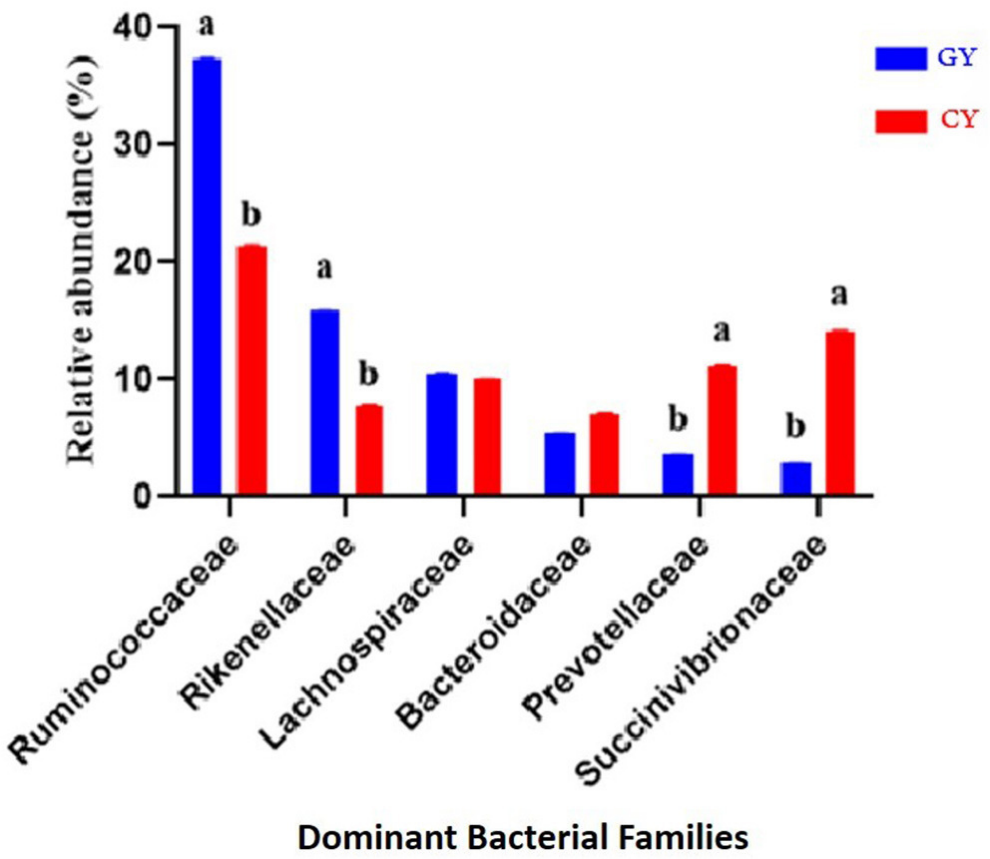
Major bacterial families found in yaks fed on natural grazing pasture (GY) and feedlots (FY). Bars with different superscripts indicate that mean values were significantly different from each other (*p* < 0.05).

At the family level, 111 families were identified in both groups, indicating 87 and 101 families in the grazing and feedlot groups, respectively. The dominant families in GY and FY are presented in Figure 5. The most abundant families in GY were *Ruminococcaceae* (37.3% ± 0.06), *Succinivibrionaceae* (2.82% ± 0.06), *Lachnospiraceae* (10.6% ± 0.04), *Rikenellaceae* (15.8% ± 0.04), *Bacteroidaceae* (5.43% ± 0.01), and *Prevotellaceae* (3.63% ± 0.01), which accounted for 75.6% ± 0.12 of the total microbial population. *Bacteroidaceae* (7.19%±0.03), *Ruminococcaceae* (21.4% ± 0.06), *Rikenellaceae* (7.76% ± 0.06), *Succinivibrionaceae* (14.0% ± 0.13), *Prevotellaceae* (11.2% ± 0.07), and *Lachnospiraceae* (10.0% ± 0.04) were identified in the FY group, accounting for 71.5% ± 0.09 of the total fecal microbiota. *Ruminococcaceae* and *Rikenellaceae* were significantly higher (*p* < 0.001) in GY than in the FY group, whereas *Prevotellaceae* and *Succinivibrionaceae* were significantly higher (*p* < 0.001) in FY than in GY. *Lachnospiraceae* and *Bacteroidaceae* did not differ (*p* > 0.05) between groups. Some other families were also detected, but their abundances were quite low.

### Relative Abundance of Bacterial Genera

A total of 203 genera were identified in the two groups in which 156 and 182 genera were found in GY and FY, respectively. The top 35 dominant genera observed in both groups are presented in Supplementary Figure 3. The most abundant genera included *Alloprevotella, Faecalibacterium, Succinivibrio*, and *Ruminobacter* in both groups. *Faecalibacterium, Alloprevotella*, and *Succinivibrio* were significantly higher (*p* < 0.001) in FY than in GY (Supplementary Figure 4), while *Bacteroides* and *Ruminobacter* did not differ (*p* > 0.05) between groups.

**Figure 6:**
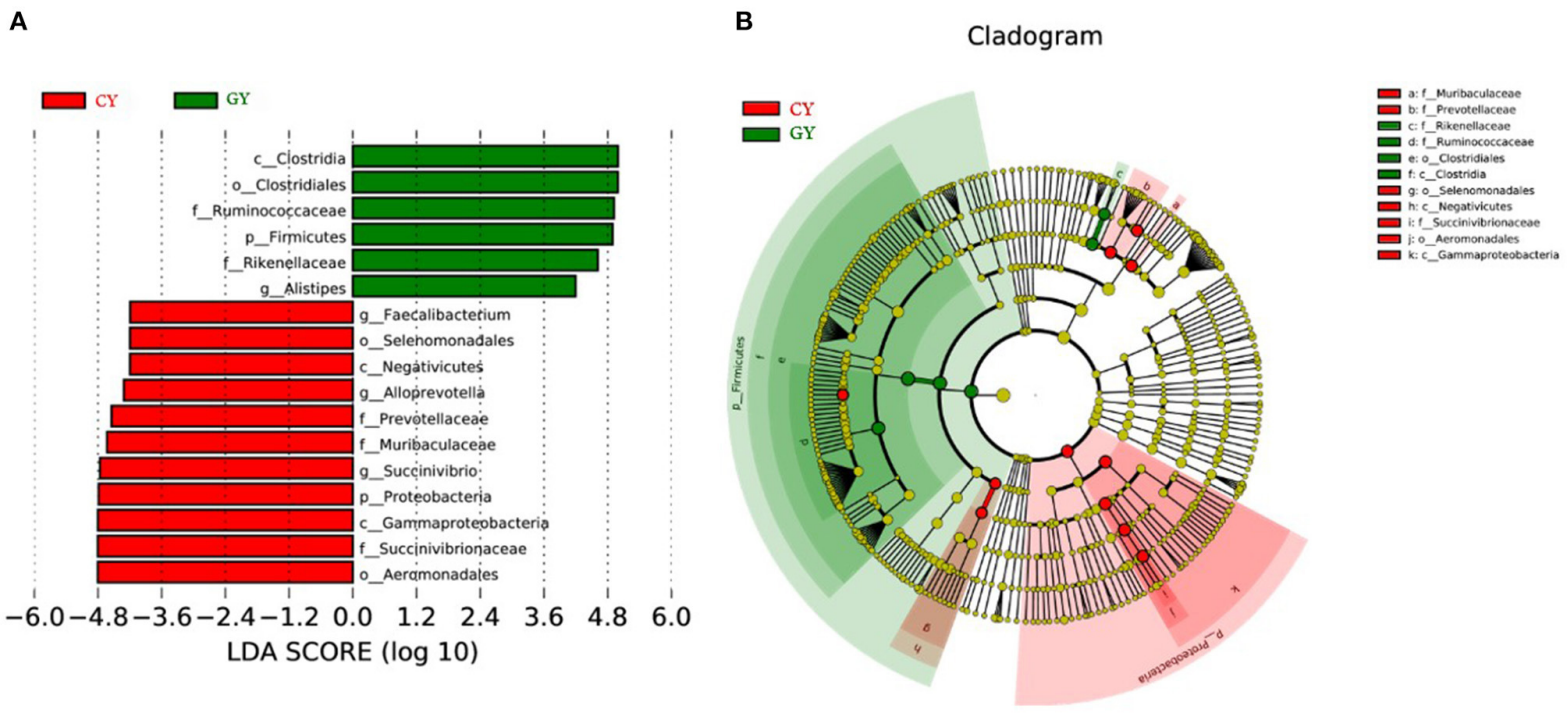
Cladogram showing differential bacterial taxa **(A)** and linear discriminant analysis (LDA) effect size (LEfSe) indicating biomarker taxa **(B)** in yaks fed on natural grazing pasture (GY) and feedlots (FY).

Great variation was observed among individual animals in prokaryotic community composition at the phylum, family, and genus levels. Linear discriminant analysis effect size (LEfSe), including LDA, was conducted to examine the differential microbial communities between GY and FY groups. The GY group samples contained *Clostridia, Ruminococcaceae*, and *Rikenellaceae*, while the FY group harbored *Gammaproteobacteria, Aeromonadales, Succinivibrionaceae, Prevotellaceae*, and *Muribaculacceae* as biomarker taxa (Figures 6A,B).

## Discussion

In the present study, the ash, CP, EE, NFC, hemicellulose, and DDM contents of forages in grazing pasture were higher than those of forages in the feedlot. The OM, hardly fermentable dietary fiber such as NDF and ADF, and TC content of forages in feedlot were higher than forages in grazing pasture. The variation in the chemical composition of forages in grazing pasture and feedlot is related to many factors such as stage of growth, maturity, species or variety, (25) drying method, growth environment (26), and soil types (27). Better nutrient composition and palatability of forage in the grazing pasture might be attributed to the higher CP content and low insoluble fiber contents as compared to that of forage in feedlot.

The alpha diversity indices (including ACE, Chao1, and Shannon) showed that the fecal bacterial diversity of GY was higher than in FY. Earlier studies have demonstrated that fiber-based diets improved microbial communities because fiber fermentation enhances microbial proliferation better than starch fermentation (28, 29). Fiber-based diets contain more secondary plant compounds, which are favorable to act as prebiotics and assist in the improvement of bacterial diversity (30, 31). In the present study, forages on natural grazing land contained 22.7% NFC in which sugar, oligosaccharide, and peptic polysaccharide were essential ingredients. Therefore, bacterial communities of yaks grazing on natural pasture showed an improvement in beta diversity. Furthermore, forage in GY was higher in hemicellulose and higher digestible dry matter and crude protein contents, which might help the proliferation of microbial community. Moreover, forage varieties and biomass may also influence the diversity of the microbial community in yaks.

The dissimilarity in the microbial community between the fecal samples of GY and FY was about 20.4 and 45.2%, respectively. The previous study reported that the dissimilarity in the microbial community between the rumen samples of yaks was approximately 24.1% (32). The relative microbial abundance and microbial diversity richness is affected by the forage quality (8) as observed in the present study. Nutrient quality and higher digestible dry matter content of forage reduced the dissimilarity of microbial diversity in the GY.

The relative abundances of microbes differed between the two yak groups. The abundances of two dominant bacterial phyla in GY were Firmicutes (56%) and Bacteroidetes (36%), and those in FY were Firmicutes (41%) and Bacteroidetes (37%). Previous studies reported that the relative abundance of Firmicutes was higher than Bacteroidetes in yak and bison under grazing conditions (33, 34), which was consistent with the present study. Other studies concluded that Bacteroidetes or Firmicutes' dominance could be attributed to variation in diet, climate, and farming practices in a wide geographical range (35). However, in a narrow geographical range, diet composition and host species had little impact on the dominant position of these two bacterial phyla (5).

Moreover, the Firmicutes-to-Bacteroidetes ratio is an important factor in assessing the effect of gut microbes on host energy requirements (36). In the present study, Firmicutes-to-Bacteroidetes ratio in GY was considerably higher (1.6:1) than in FY (1.1:1). Therefore, yaks fed on natural grazing get more energy content, which is required for metabolism. Studies have reported that the ratio of Firmicutes to Bacteroidetes is related to roughage proportion and milk-fat yield (37). According to previous findings, the proportions of Firmicutes in sheep of the Qinghai-Tibetan Plateau (QTP) were higher than in lowland sheep and goats. Gram-positive bacteria play an important role in the digestion of specific grasses available at the QTP (34). These findings are consistent with the present study as the different locations and different diets might have affected the abundance of Firmicutes in GY and FY. Higher abundance of Firmicutes in this study might be associated with factors, such as age (38), location (34), and diet (39). The environment, forage quality, and forage varieties in grazing pasture seem more favorable for Firmicutes proliferation. Previous studies have also reported that starch and fat-rich high-energy diets increased Firmicutes abundance (40). In the present study, the forages in grazing pasture may have increased soluble fiber and starch content because of high protein content and low fermentable fiber contents. Similar studies have reported that the most abundant microbial phyla in the grazing yak rumen in QTP were Firmicutes, Bacteroidetes, and Proteobacteria (5, 13, 34). These findings are in line with the present study as we observed dominancy of Firmicutes, Bacteroidetes, and Proteobacteria in GY and FY. Results of the present study indicated that the abundances of Bacteroidetes, Firmicutes, and Proteobacteria were influenced by diet and environment as reported previously (34, 39).

The most abundant families in both yak groups were Ruminococcaceae, Succinivibrionaceae, Lachnospiraceae, Rikenellaceae, Bacteroidaceae, and Prevotellaceae, with Ruminococcaceae and Rikenellaceae being higher in GY than in FY. These bacteria play a vital role in starch and fiber degradation and improvement of fiber digestibility (41). Similarly, Ruminococcaceae have been reported to degrade protein (42). The availability of high-quality forages and adequate nutrients in grazing pasture favored the relative abundance of fiber-degrading bacteria such as Ruminococcaceae and Rikenellaceae. Lachnospiraceae was found in Holstein cows' rumen, and it plays an important role in the growth stimulation of fibrolytic bacteria (43, 44). In the present study, Prevotellaceae and Succinivibrionaceae in FY were higher than in GY. Prevotellaceae is one of the dominant bacteria of the saccharolytic group in the rumen and is known for its protein binding ability and digestion of multiple carbohydrate substrates (45). The relatively higher abundance of Prevotellaceae in the feces of the yaks indicated high carbohydrate degradation ability attributed to the high OM content in the feed. Two recent studies reported that Christensenellaceae, Ruminococcaceae, Rikenellaceae, and Prevotellaceae played important roles in forage degradation in the rumen since these groups tightly adhere to forage grass after incubation in the rumen (46, 47). In the present study, a higher proportion of Ruminococcaceae and Rikenellaceae in GY is expected to improve fiber degradation.

Among dominant genera, *Bacteroides, Succinivibrio, Ruminobacter, Alloprevotella*, and *Faecalibacterium* were the most abundant in GY and FY. *Succinivibrio* is a starch-degrading bacteria that produces mainly acetate and succinate. *Ruminobacter* is important for starch digestion and fiber degradation in the rumen. *Prevotella*, belonging to Alloprevotella, has been characterized by large genetic divergence and possesses functional versatility (48), which is essential for initial dietary protein breakdown (49), peptide metabolism (50), starch degradation, and efficient utilization of hemicelluloses (51). Alloprevotella might also possess the same functional versatility as *Prevotella. Succinivibrio, Ruminobacter*, and *Alloprevotella* enhance fiber-degrading bacteria. *Bacteroides* are a dominant bacterial genus in the intestinal microbial community of diarrheal yaks that absorb nutrition and produce short-chain fatty acid (SCFA) (52), restoring and promoting the maturation of epithelial cells related to the metabolism of fat. *Faecalibacterium* could protect from inflammation (53) and generate butyrate and encourage the maintenance of intestinal mucosa (54). *Bacteroides* encode multiple proteins and transport complex carbohydrates and possess higher carbohydrate-degrading ability (45). *Bacteroides* and *Faecalibacterium* aid in the production of SCFA, which assist in maintaining the intestinal epithelium, and balancing the microbial community structure in yaks. In the present study, *Faecalibacterium, Alloprevotella*, and *Succinivibrio* were higher in FY than in GY. *Faecalibacterium* and *Succinivibrio* increased SCFA production and microbial proliferation. *Prevotella* belongs to *Alloprevotella* capable of metabolizing dietary fibers from plant cell walls, thus producing significant amounts of SCFAs (55). *Alloprevotella* might enhance dietary fiber utilization like *Prevotella*. These findings might be attributed to the nutrient composition of forages in the feedlot as higher OM contents favored the proliferation of these fibrolytic bacteria.

The environment, forage quality, and forage varieties could be related to microbial composition, diversity, and function of the bacteria in the feces of yak. Bacterial composition and diversity richness might be improved among domestic yaks under grazing at natural pastures when high-quality forages, adequate nutrient, and forage biomass in grazing pasture are available. However, when forage quality declines, supplementation of total mix ration in the diet would be required to maintain energy homeostasis and optimum microbial diversity.

## Conclusion

The findings of the present study indicated that grazing of yaks on natural pastures increased the diversity of bacterial communities in fecal microbiota. Moreover, the relative abundance of Firmicutes was higher in yaks that grazed on natural pastures as compared to the confined yaks. Grazing on natural pastures favored the fiber-degrading bacteria (belonging to Ruminococcaceae and Rikenellaceae) while feedlot feeding improved the abundance of protein- and carbohydrate-degrading bacteria (Prevotellaceae) in yak. These observations contribute novel insights into the current understanding of yak gut microbiota and provide evidence for the effect of type of forage on the rumen microbiota.

## Data Availability Statement

The datasets presented in this study can be found in online repositories. The names of the repository/repositories and accession number(s) can be found at: https://www.ncbi.nlm.nih.gov/, PRJNA633755.

## Ethics Statement

The animal study was reviewed and approved by the Ethics Committee of School of Life Sciences, Lanzhou University, Lanzhou, China.

## Author Contributions

TS and LD: conceptualization. TS: methodology, investigation, and writing—original draft preparation. HW: software. AU and AA: validation. XW: formal analysis. QY and YF: resources. F-uH: data curation. F-uH, NA, and AU: writing—review and editing. HW and XW: visualization. LD: supervision, project administration, and funding acquisition. All authors have read and agreed to the published version of the manuscript.

## Funding

This research was funded by the National Key R&D Program of China (2021YFD1300500), the Qinghai Provincial Science and Technology Major Project 2021-NK-A5, and Platform of Adaptive Management on Alpine Grassland-livestock System [2020-ZL-T07].

## Conflict of Interest

The authors declare that the research was conducted in the absence of any commercial or financial relationships that could be construed as a potential conflict of interest.

## Publisher's Note

All claims expressed in this article are solely those of the authors and do not necessarily represent those of their affiliated organizations, or those of the publisher, the editors and the reviewers. Any product that may be evaluated in this article, or claim that may be made by its manufacturer, is not guaranteed or endorsed by the publisher.
