# Supplementary material for: Differential Effects of Natural Grazing and Feedlot Feeding on Yak Fecal Microbiota
Source: Front Vet Sci. 2022 Apr 15;9:791245. doi: 10.3389/fvets.2022.791245 (PMC9074760; doi:10.3389/fvets.2022.791245)
Supplement: Supplementary file 1 [file Data_Sheet_1.docx]

**Supplementary Material**

Differential effects of natural grazing and feedlot feeding on Yak fecal microbiota


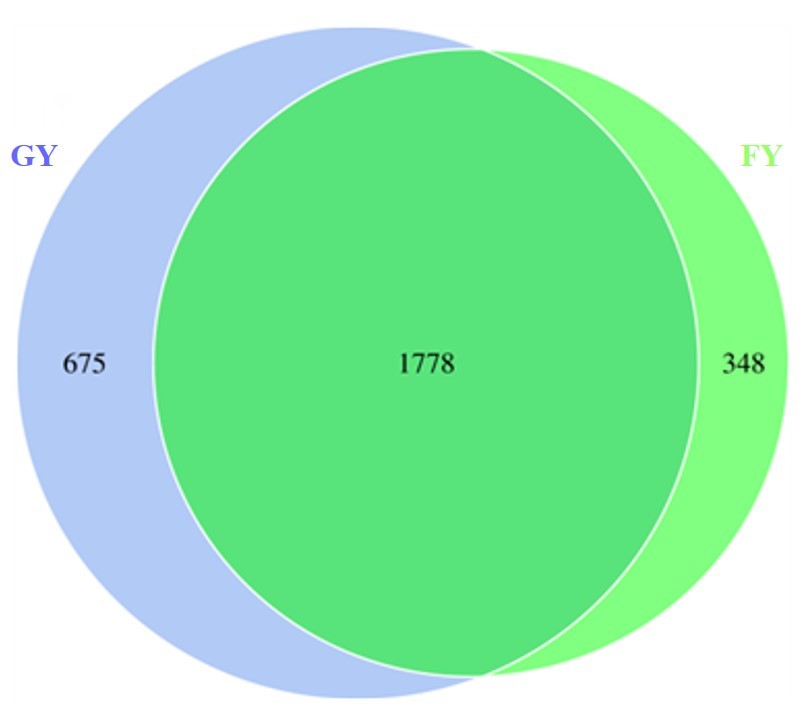


Figure S-1. VENN diagram showing number of shared and unique OTUs in both Yak groups. GY: Yaks in grazing group; FY: Yaks in feedlot group


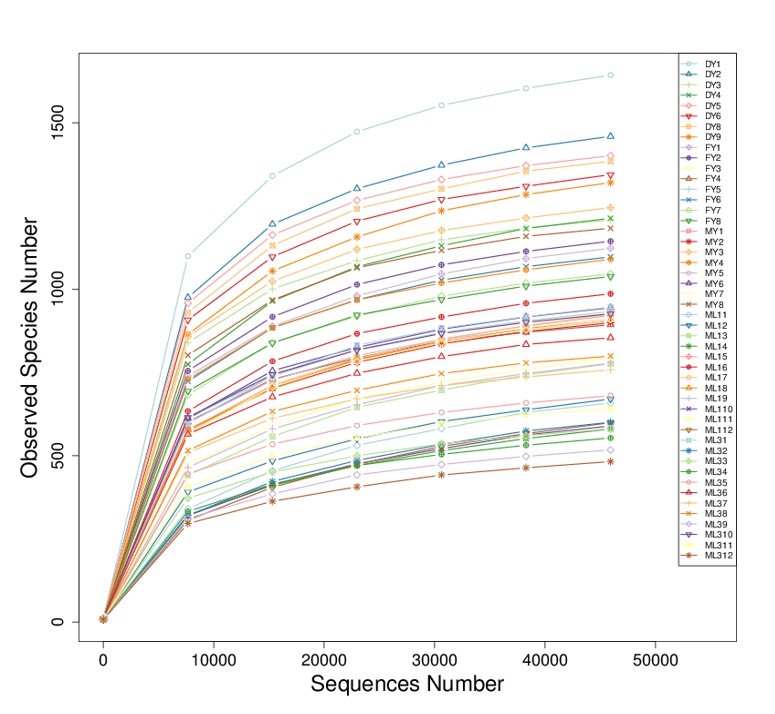


Figure S-2. Rarefaction curves of showing species richness and sequencing depth in both groups. GY:Yaks in grazing group; FY:Yaks in feedlot group


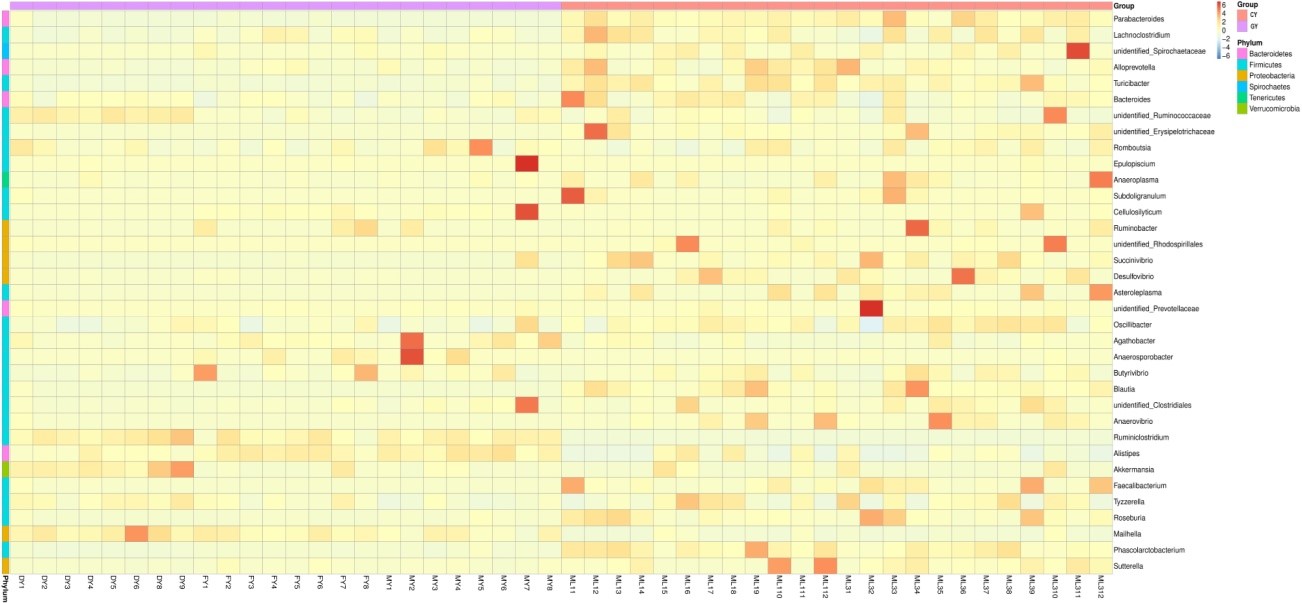


Figure S-3. Heat map analysis of top 35 bacterial genera observed among Yaks fed on natural grazing pasture (GY) and feedlot (FY).


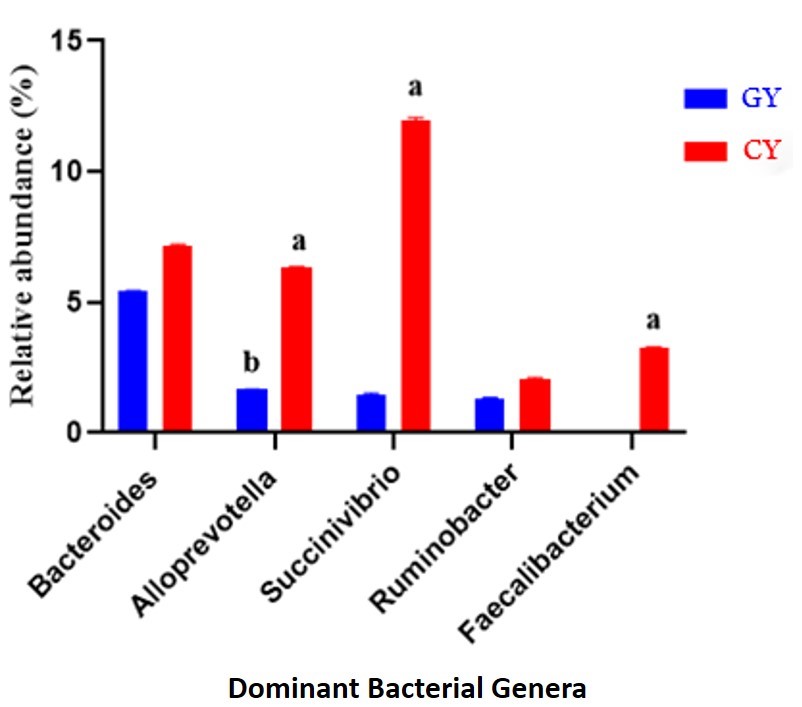


Figure S-4. Dominant bacterial genera observed in yaks fed on natural grazing pasture (GY) and feedlot (FY)
